# Elevated TMEM106B levels exaggerate lipofuscin accumulation and lysosomal dysfunction in aged mice with progranulin deficiency

**DOI:** 10.1186/s40478-017-0412-1

**Published:** 2017-01-26

**Authors:** Xiaolai Zhou, Lirong Sun, Owen Adam Brady, Kira A. Murphy, Fenghua Hu

**Affiliations:** 1 Department of Molecular Biology and Genetics, Weill Institute for Cell and Molecular Biology, Cornell University, Ithaca, 14853 NY USA; 2 Department of Neurobiology, School of Basic Medical Sciences, Southern Medical University, Guangzhou, China

**Keywords:** Frontotemporal lobar degeneration (FTLD), Progranulin, TMEM106B, Lipofuscin, Lysosome

## Abstract

Mutations resulting in haploinsufficiency of progranulin (PGRN) cause frontotemporal lobar degeneration with TDP-43-positive inclusions (FTLD-TDP), a devastating neurodegenerative disease. Accumulating evidence suggest a crucial role of progranulin in maintaining proper lysosomal function during aging. TMEM106B has been identified as a risk factor for ﻿frontotemporal lobar degeneration ﻿with progranulin mutations and elevated mRNA and protein levels of TMEM106B are associated with increased risk for frontotemporal lobar degeneration. Increased levels of TMEM106B alter lysosomal morphology and interfere with lysosomal degradation. However, how progranulin and TMEM106B interact to regulate lysosomal function and frontotemporal lobar degeneration﻿ (FTLD) disease progression is still unclear. Here we report that progranulin deficiency leads to increased TMEM106B protein levels in the mouse cortex with aging. To mimic elevated levels of TMEM106B in frontotemporal lobar degeneration﻿ (FTLD) cases, we generated transgenic mice expressing TMEM106B under the neuronal specific promoter, CamKII. Surprisingly, we found that the total protein levels of TMEM106B are not altered despite the expression of the TMEM106B transgene at mRNA and protein levels, suggesting a tight regulation of TMEM106B protein levels in the mouse brain. However, progranulin deficiency results in accumulation of TMEM106B protein from the transgene expression during aging, which is accompanied by exaggerated lysosomal abnormalities and increased lipofuscin accumulation. In summary, our mouse model nicely recapitulates the interaction between progranulin and TMEM106B in human patients and supports a critical role of lysosomal dysfunction in the frontotemporal lobar degeneration﻿ (FTLD) disease progression.

**Electronic supplementary material:**

The online version of this article (doi:10.1186/s40478-017-0412-1) contains supplementary material, which is available to authorized users.

## Background

Frontotemporal lobar degeneration (FTLD) is a devastating neurodegenerative disease that affects approximately 250,000 people in the United States [24, 28]. Clinical symptoms of FTLD include behavioral abnormalities, personality changes, and language impairments [25]. A major type of FTLD shows TDP-43 and ubiquitin positive inclusions (FTLD-TDP) [3, 26]. Progranulin (PGRN) haploinsufficiency due to mutations in the *Progranulin gene (GRN)* is one of the major causes of FTLD-TDP [4, 11, 17].

Additional environmental or genetic factors influence the manifestation of FTLD-TDP, resulting in a high variability in age of onset and pathological presentation, even with identical mutations [42]. Recent genome-wide association studies by several groups have identified *TMEM106B*, a gene encoding a type II transmembrane protein of unknown function, as a bona fide risk factor for FTLD-TDP, especially in patients with PGRN mutations [10, 13, 15, 41, 43]. The TMEM106B risk allele was reported to increase TMEM106B mRNA levels [41]. Both mRNA and protein levels of TMEM106B are elevated in FTLD-TDP patients, especially in PGRN mutant carriers [7]. These data suggest that PGRN might regulate TMEM106B protein homeostasis and elevated TMEM106B levels increase the risk for FTLD-TDP with PGRN mutations.

Our recent studies and others showed that TMEM106B is highly expressed in neurons, mainly localizes in the late endosome/lysosome compartments, and regulates lysosomal morphology [6, 7, 20, 21, 36]. Overexpression of TMEM106B results in accumulation of enlarged lysosomes and delays the degradation of endocytic cargoes, such as EGFR [6]. More recently, TMEM106B was shown to interact with MAP6 to control lysosomal trafficking in dendrites and decreased levels of TMEM106B result in defects in lysosome size, mobility and lysosomal trafficking in neurons [33, 36]. These observations strongly argue that proper regulation of TMEM106B levels is critical for normal lysosomal function and that impaired lysosomal function due to elevated TMEM106B levels might accelerate the development of FTLD phenotypes. Along this line, several recent studies have suggested a critical role of PGRN in lysosomes. Human patients with a total loss of PGRN exhibit neuronal ceroid lipofuscinosis (NCL) [35], a lysosomal storage disease characterized by the accumulation of auto-fluorescent storage material (lipofuscin). NCL phenotypes and lysosomal abnormalities were also seen in PGRN knockout mice [1, 18, 38]. Furthermore, PGRN is transcriptionally co-regulated with a number of essential lysosomal genes [5], and we have demonstrated that PGRN is a lysosomal resident protein delivered to lysosomes through two independent mechanisms [19, 46]. Finally, FTLD-TDP/PGRN patients also exhibit typical pathological features of NCL pathology [18], suggesting FTLD and NCL caused by PGRN mutations are pathologically linked. Thus the identification of TMEM106B as a risk factor for FTLD with PGRN mutations and the fact that increased TMEM106B levels impair lysosomal function further underscore lysosomal dysfunction as one of the disease mechanisms for FTLD.

To determine an in vivo effect of elevated TMEM106B levels in mouse models, we generated transgenic lines expressing human TMEM106B under a neuronal specific promoter. To our surprise, we found that TMEM106B protein levels are tightly regulated despite the expression of the transgene at RNA and protein level. However, elevated TMEM106B levels were detected in aged PGRN deficient mice expressing the transgene, which exacerbates the lysosomal abnormality and lipofuscin accumulation in the PGRN deficient background. Thus our data nicely illustrates the cross-regulation between PGRN and TMEM106B during aging and neurodegeneration.

## Methods

### Pharmacological reagents and antibodies

The following antibodies were used in this study: rat anti-mouse LAMP1 (1D4B) from BD Biosciences, goat anti-mouse Cathepsin D (C-20) from Santa Cruz and mouse anti-beta III tubulin from Promega. Rabbit anti-subunit c of mitochondrial ATP synthase (SCMAS) antibodies [32] were a gift from Dr. Elizabeth F. Neufeld (David Geffen School of Medicine, University of California, Los Angeles, CA). Rabbit anti-TMEM106B antibodies were generated against the cytosolic domain of human TMEM106B as previously described [6].

### Mouse strains

Human TMEM106B cDNAs with polyA sequence from the pEGFP-C1 vector were amplified and cloned into the pMM403 plasmid from Addgene (plasmid 34926) using the NotI restriction site. The expression cassette was excised by digestion with the restriction enzyme SfiI and injected into the pronuclei of fertilized eggs derived from FVB/NJ strain by the Transgenic Mouse Facility at Cornell University to yield offspring with different expression levels of TMEM106B. 16 pups were born after one round of injection and implantation. The founder #2 with the highest TMEM106B was selected and back crossed with wild type C57/BL6 for three generation for experimental analysis. Wild type C57/BL6 and *PGRN−/−* mice were obtained from Jackson Laboratories. All mice were housed in the Weill Hall animal facility.

### Genotyping

Genomic DNA was extracted from mouse tails using the REDextract kit from Sigma. Primers with sequences 5’TCCAACCCCCTCAGTACATC3’ and 5’TTTTCTTGCCCCCTAGGAAT3’ were used to identify human TMEM106B transgene (594 bp PCR product). Notch primers (5’ GATATC GTGGTGCATACCCTCCTG3’ and 5’ GTGGTCTAGGATGCTTGGGTCTAG 3’) were used to amplify Notch1 as an internal control (300 bp PCR product). Progranulin deficient mice were genotyped using the mixture of following primers: 5’ AGAGGGTGAGCTGCAATGTT 3’, 5’AAGGGCATTAGCCAAGTGTG3’ and 5’TCTCCCAGGTAGCCCCTACT3’ in which wild type has a 468 bp product and *PGRN−/−* has a 211 bp PCR product.

### Western blot analysis

Cortices were homogenized in RIPA buffer on ice and an equal volume of 2X SDS sample buffer was added before sonication. Samples were maintained on ice throughout before loading on SDS-PAGE. TMEM106B protein runs as a dimer under this condition (Additional file 1) [7]. Western blots were done as previously described [6]. TMEM106B protein levels were quantified using LiCor Odyssey system and normalized to beta III tubulin.

### qPCR analysis

mRNAs were extracted from cortices using TRIZOL (Invitrogen). mRNAs were reversed transcribed to cDNAs using iScript kit (Biorad). Real time PCRs were done on Roche Lifecycler 480 using the following primers: mouse actin (5’ACGAGGCCCAGAGCAAGAG3’ and 5’TCTCCAAGTCGTCCCAGTTG3’), mouse TMEM106B 5’CGCGTGCGGTTTCTAGAGCAT 3’ and 5’CCTCCCCGGGCTCTCAATGT3’) and human TMEM106B (5’GGGCAAGAAAACCAACTGGTGGC3’ and 5’ TCACGTCGATAGAGCGAGGGAA 3’). All the primers have the amplification efficiency close to 100%. Transcript levels were calculated using efficiency-adjusted ΔΔ-CT. All transcripts were normalized to actin.

### Mass spectrometry analysis

Cortices were dissected from TMEM106B transgenic mice at 1.5 months old and lysed in 50 mM Tris, 150 mM NaCl and 1% Triton plus protease inhibitors (Roche). After centrifugation, the supernatant was immunoprecipitated with Affigel (Biorad) conjugated with anti-TMEM106B antibodies. 2% of the IP products were eluted, trypsinized and analyzed by mass spec as previously described [19, 46].

### Immunofluorescence microscopy

Mouse brains were perfused and fixed with 4% formaldehyde. After gradient dehydration with 15% and 30% sucrose, the mouse brains were embedded with OCT compound (Sakura Finetek USA) and sliced with Cryotome. For immunostaining (not for lipofuscin analysis), brain sections were incubated with 0.01% Sudan Black B (Spectrum Chemical) in 70% ethanol at room temperature for 20 min to negate the autofluorescence, then permeabilized and blocked in blocking buffer (0.05% saponin, 3% BSA in TBS) for 1 h. Primary antibodies were incubated in blocking buffer overnight at 4 °C. Sections were washed and incubated in secondary antibodies conjugated to Alexaflour 488, 568, or 660 (Invitrogen). Sections were washed three more times and coverslips mounted onto slides with Fluoromount G (Southern Biotech). Images were acquired on a CSU-X spinning disc confocal microscope (Intelligent Imaging Innovations) with an HQ2 CCD camera (Photometrics) using a 40x or 100x objective.

### Quantification of enlarged lysosomes and lipofuscin

For the quantification of enlarged lysosomes, the lysosomes were visualized by anti-LAMP1 staining, and the entire neuron somas were captured using Z stack. Neurons with enlarged lysosomes (diameter > 1.0 μm) were counted. It should be noted that during fixation, lysosomal size and area might have been changed but we always have a control group and experimental group analyzed at the same time. For lipofuscin analysis, brain sections were stained with Hochest 33324 solution (Thermo Fisher Scientific) after permeabilization and blocking. Images were acquired on an epifluorescence microscope (Zeiss) equipped with a CCD camera. Auto fluorescent signals were quantified using Image J (NIH).

### Statistical analysis

The data were presented as mean ± SEM. Statistical significance between multiple groups was analyzed by one-way ANOVA followed by Bonferroni’s multiple comparison test. Two-group analysis was performed using the Student’s t test. *P*-values <0.05 were considered statistically significant. All statistical analyses were performed using GraphPad Prism 5 software (GraphPad Software).

## Results

### Generation of TMEM106B transgenic mice

**Fig. 1:**
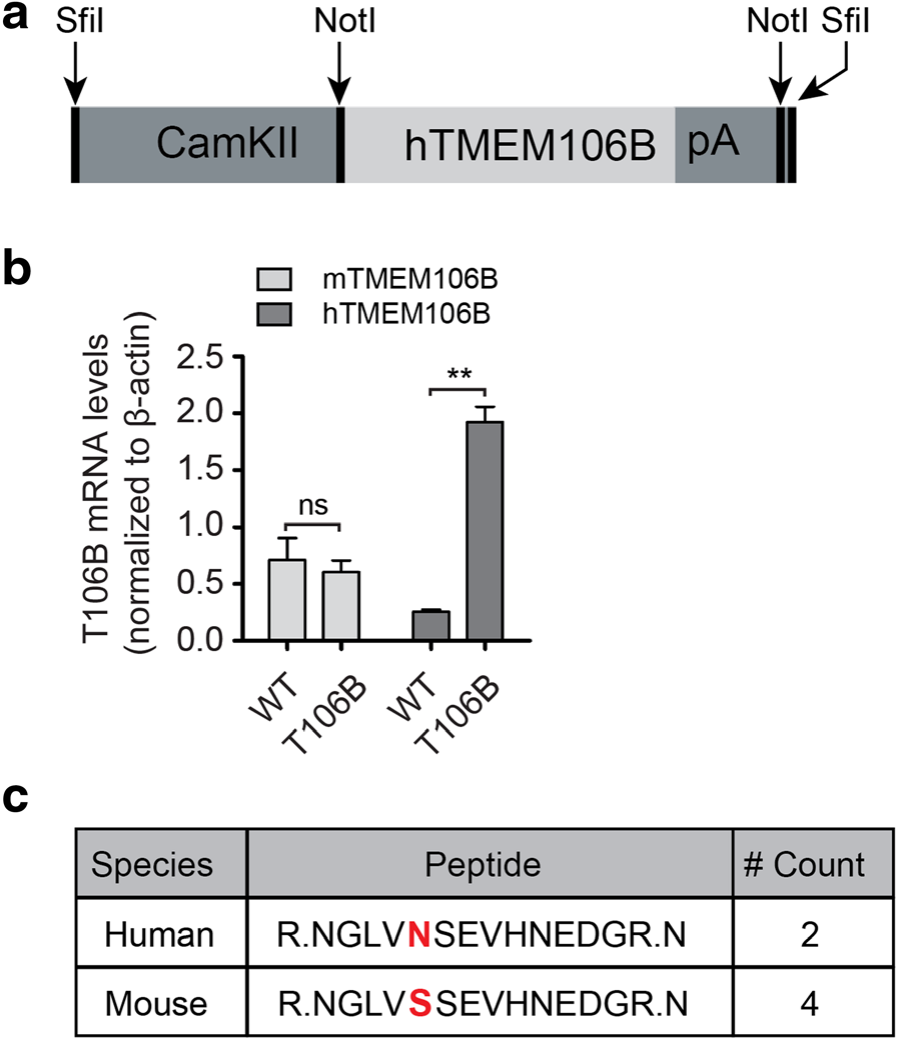
Generation of TMEM106B transgenic mice. **a** Schematic drawing of the expression construct used to inject the pronuclei of fertilized eggs. Human TMEM106B and poly A sequence were cloned into the NotI site of pMM403 and the cassette was excised with SfiI for injection. **b** qPCR analysis of mouse TMEM106B and human TMEM106B mRNA levels in the offspring of highly expressed transgenic line and WT littermate controls of 4–5 months of age. Relative mRNA levels are normalized to actin. *n* = 3, student’s t-test, *, *p* < 0.01. **c** Mass spectrometry analysis of TMEM106B from the transgenic line. TMEM106B protein was immunoprecipitated from the cortical lysates of the transgenic line and the IP product was trypsin digested and subject to mass spec analysis

Since elevated TMEM106B levels increase the risk of FTLD with PGRN mutations, transgenic mice with TMEM106B overexpression would allow us to understand the consequences of TMEM106B overexpression in animal models. Our preliminary data suggest that TMEM106B is highly expressed in neurons and that overexpression of TMEM106B in neuronal cell lines results in enlarged lysosomes and lysosomal dysfunction [6]. Given these preliminary findings, we generated constructs expressing human TMEM106B cDNA under the neuron specific promoter, CaMKII alpha [23] (Fig. 1a). Our previous studies have shown that fusion of an epitope tag on TMEM106B often leads to changes in TMEM106B localization and function [6]. Thus we decided to express a human TMEM106B transgene without an epitope tag. The expression cassette was injected into the pronuclei of fertilized eggs derived from an FVB/NJ strain. Initial screening using primers specific for the transgene identified 3 positive pups containing different copy numbers of the transgenic construct (data not shown). These pups were further crossed with wild type C57/BL6 and the expression of transgenes was analyzed by real time PCR in the cortex of offspring at weaning age when the CamKII promoter is active [23]. For the rest of the study, we focused on the highest expression line #2. When normalized to actin, the human TMEM106B gene appears to be expressed at mRNA level close to 3 fold that of endogenous mouse TMEM106B in line #2 (Fig. 1b). The expression of human TMEM106B transgene does not seem to affect the RNA levels of endogenous mouse TMEM106B (Fig. 1b). Due to the high sequence similarity between mouse and human TMEM106B (Additional file 2), our home made anti-human TMEM106B antibodies also recognize mouse TMEM106B. To examine whether human TMEM106B protein is expressed in the transgenic lines, we immunoprecipitated (IP) TMEM106B from the cortical lysates of transgenic TMEM106B mice and determined the presence of human specific peptides by mass spectrometry. Human specific peptides are clearly detectable in trypsin digested IP products from the transgenic lines (Fig. 1c), suggesting that the human transgene is expressed at both mRNA and protein levels.

### Tight regulation of TMEM106B protein levels in the transgenic line

**Fig. 2:**
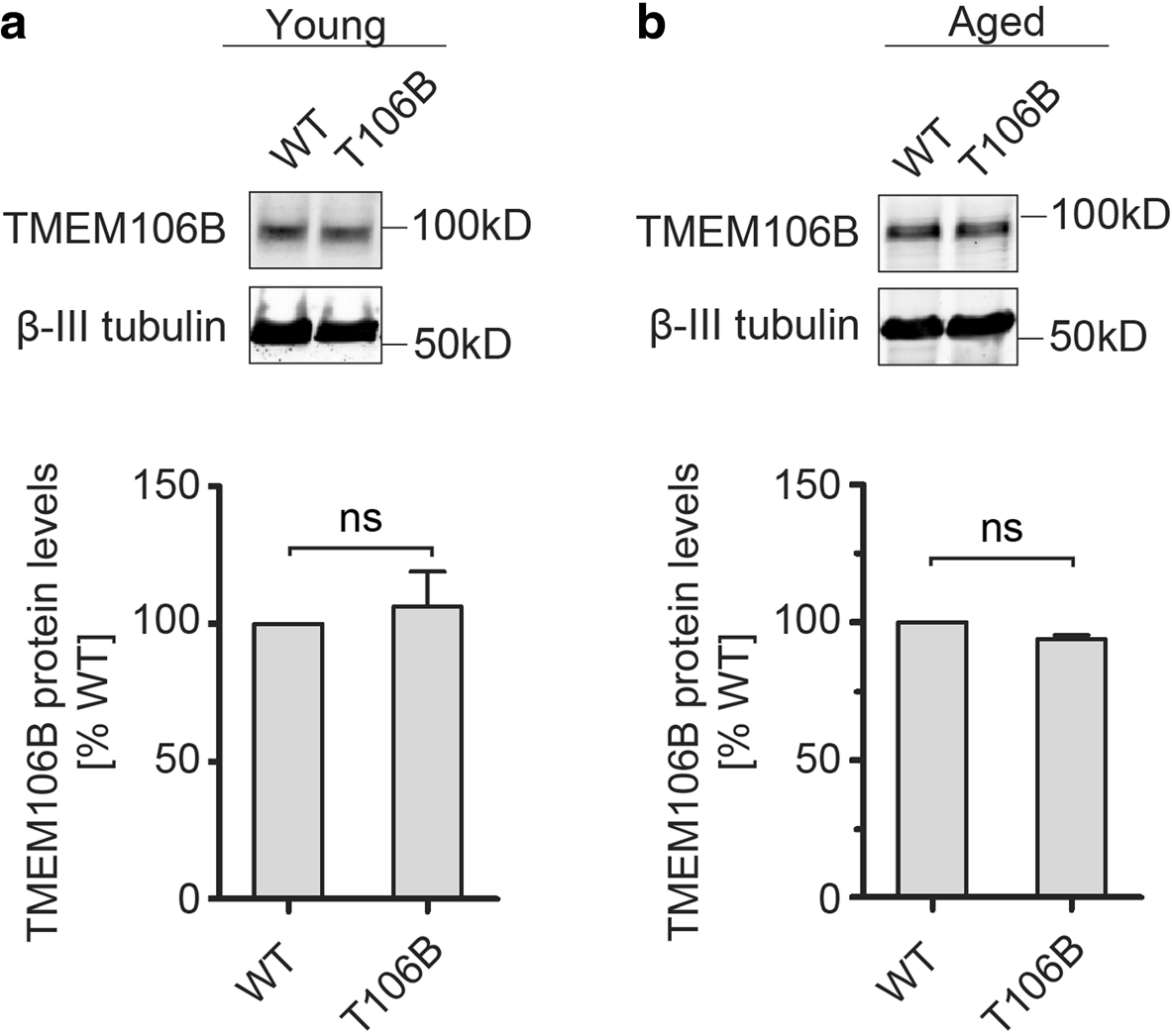
TMEM106B protein levels are tightly regulated in the mouse cortex. (**a**, **b**) TMEM106B protein levels are not changed in the transgenic mice of 4–5 months of age (**a**) or 17-20months of age (**b**). Cortical lysates from the transgenic and littermate controls were subjected to Western blot with anti- TMEM106B antibodies. n = 3, student’s t-test. ns, no significance

Next we determined the TMEM106B protein levels in the transgenic line using our polyclonal anti-human TMEM106B antibodies which recognize both human and mouse TMEM106B. Despite expression of the TMEM106B transgene at both mRNA and protein levels (Fig. 1b and c), we failed to detect an increase of total TMEM106B protein levels in the transgenic line (Fig. 2a). Aging is the biggest risk factor for neurodegenerative diseases, including FTLD. Lysosomal activities, which regulate TMEM106B turnover [6, 7, 21], are known to decline during aging. Therefore we asked whether we can detect increased TMEM106B protein levels in the aged brain in the transgenic mice compared to littermate controls. Again, we failed to see any increase in TMEM106B protein levels in the aged transgenic mice (Fig. 2b). These results strongly suggest that the level of TMEM106B protein is tightly regulated in both young and aged mouse brain.

### Progranulin deficiency leads to increased TMEM106B levels in the aged mice

**Fig. 3:**
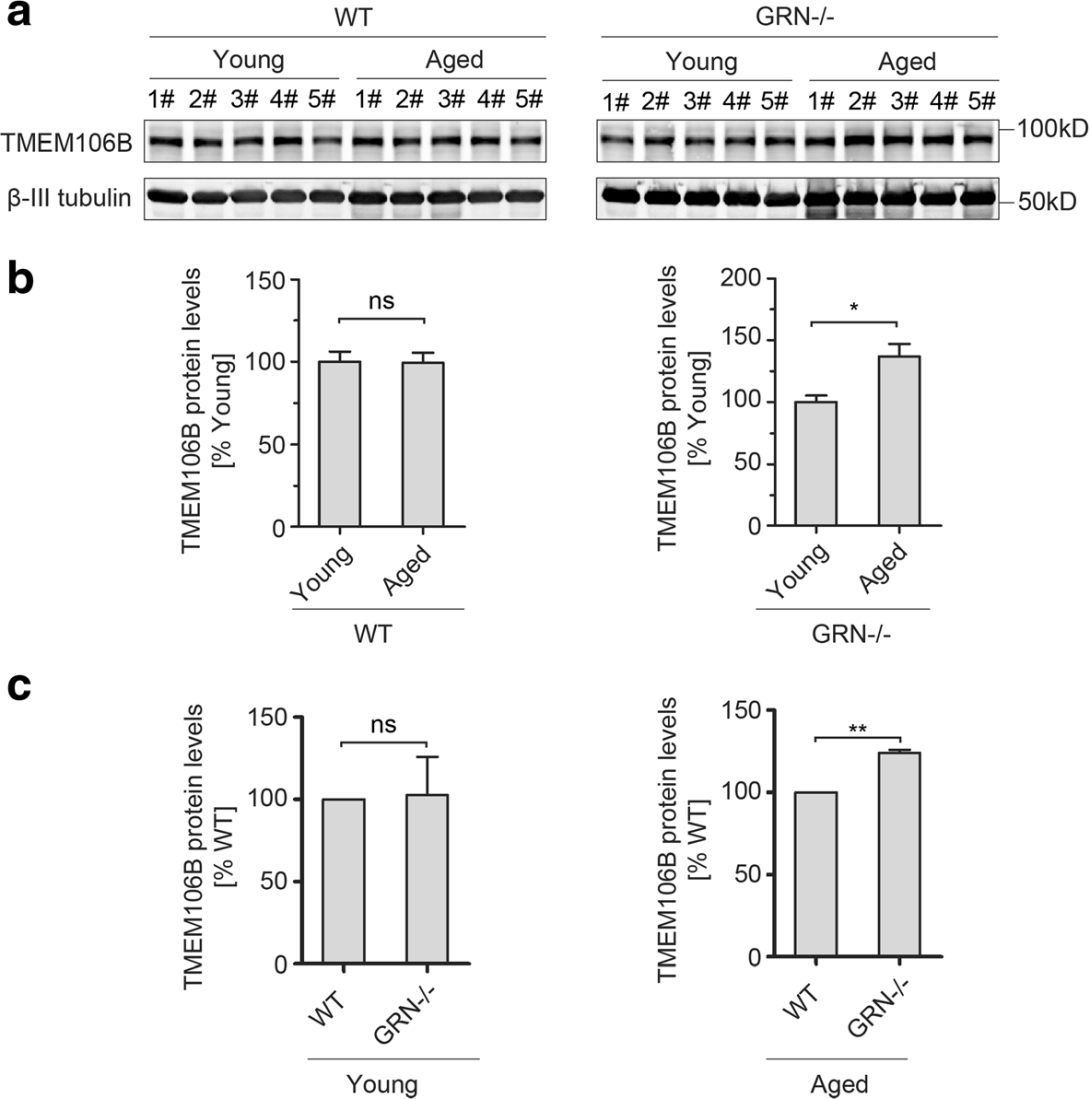
Regulation of TMEM106B protein levels by PGRN in aged brain. **a** Endogenous TMEM106B protein levels are elevated in PGRN−/− mice but not in wild type mice upon aging. Cortical lysates from mice of indicated genotypes and ages (young: 4–5 months; aged: 17–20 months old) were subjected to Western blot with anti- TMEM106B antibodies. **b** Quantification of (**a**) to compare TMEM106B protein levels between young and aged mice in the WT (*left*) or GRN−/− (*right*) background. **c** Quantification of TMEM106B protein levels between WT and GRN−/− mice during young or aged conditions. *n =* 3-5, student’s t-test. *, *p <* 0.05; **, *p <* 0.01. ns, no significance

Since TMEM106B was first identified as a risk factor specific for PGRN mutant carriers [10, 15, 41, 43], we determined whether PGRN regulates the homeostasis of TMEM106B protein levels during aging. TMEM106B protein levels in the cortex do not show any detectable changes with aging in the wild type mice (Fig. 3a), but PGRN deficiency leads to significant increase of cortical TMEM106B protein levels with aging (Fig. 3a, b). Consistent with this result, TMEM106B protein levels in the cortex do not differ in the age matched young wild type or PGRN−/− mice (4–5 month old) (Fig. 3c) but are significantly increased in aged PGRN−/− mice compared to wild type controls (Fig. 3c), suggesting a role of PGRN in maintaining TMEM106B homeostasis during aging. Previously, PGRN deficiency has been reported to result in increased TMEM106B protein levels in both young and old mice [18]. Although the cause of the discrepancy is not clear, it might be explained by strain difference.

**Fig. 4:**
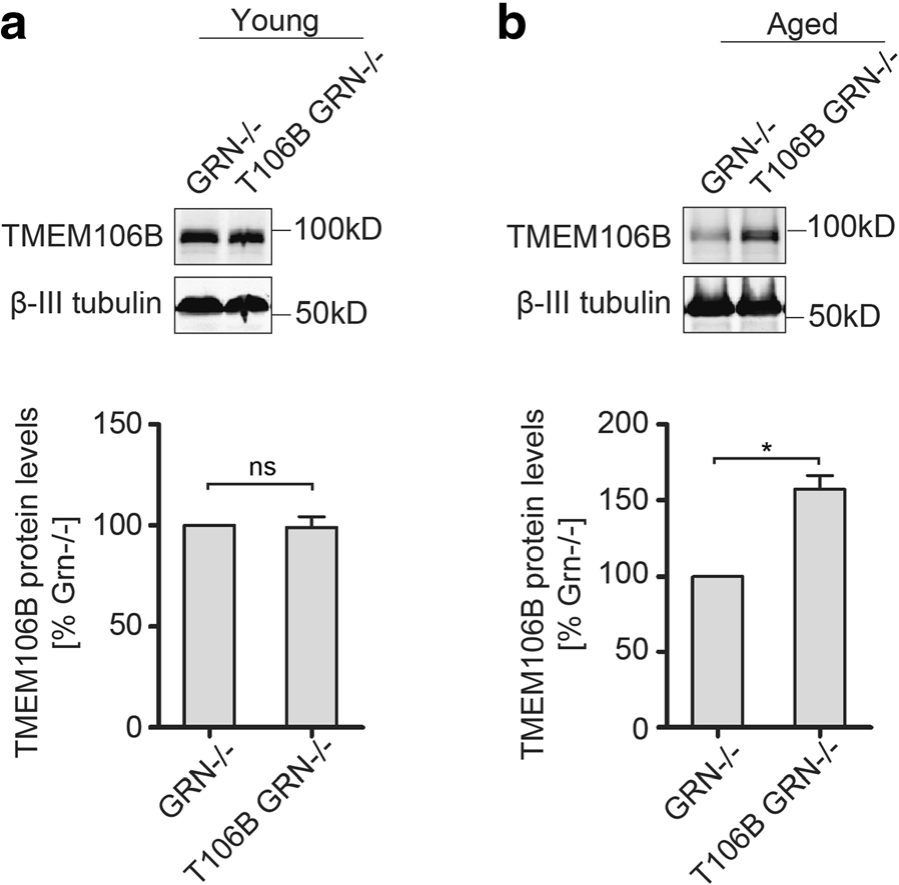
Expression of the TMEM106B transgene results in elevated TMEM106B levels in aged PGRN−/− mice. **a** Transgene expression does not lead to changes in TMEM106B protein levels in young PGRN−/− mice of 4–5 months old compared to littermate WT controls. Cortical lysates from the transgenic and littermate controls were subjected to Western blot with anti- TMEM106B antibodies. **b** TMEM106B transgene expression leads to increased TMEM106B protein levels in aged PGRN−/− mice (17-20 months old). *n =* 3, student’s t-test. *, *p <* 0.05; ns, no significance

Next, we examined the levels of TMEM106B protein in PGRN−/− mice expressing the TMEM106B transgene. While expression of the TMEM106B transgene did not lead to an increase in TMEM106B protein levels in the cortex of young adult mice with PGRN deficiency (Fig. 4a), a significant increase in the TMEM106B protein levels was detected in aged PGRN−/− mice expressing the transgene compared to PGRN−/− controls in aged brain (Fig. 4b). This strongly argues for a regulation of TMEM106B protein homeostasis by PGRN in the aged brain.

### Expression of TMEM106B transgene exacerbates lysosomal abnormalities of PGRN deficient mice

**Fig. 5:**
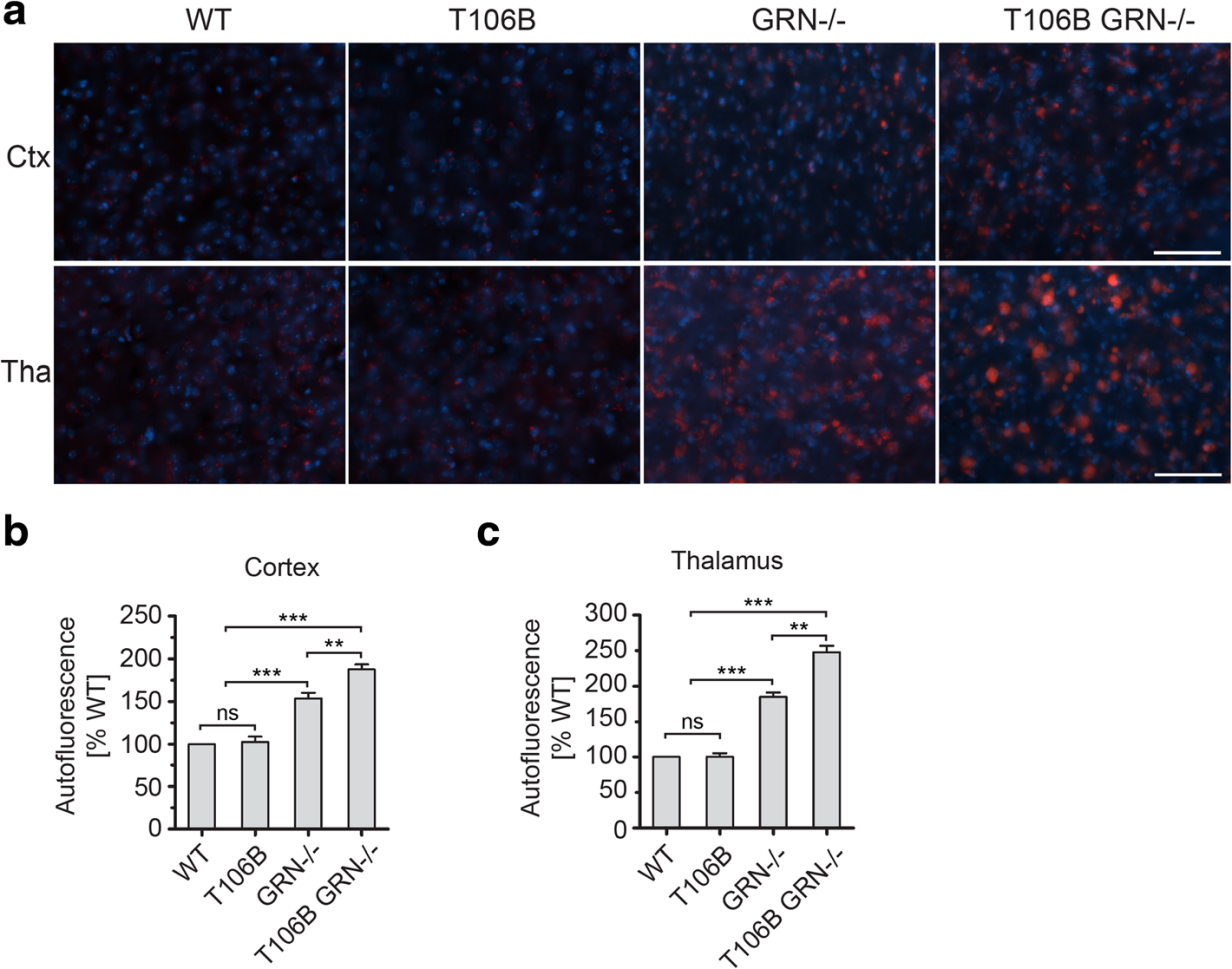
TMEM106B transgene expression increases lipofuscin accumulation in PGRN deficient mice. **a** TMEM106B transgene expression results in increased autofluorescence in the cortex and thalamus in PGRN-/- mice but has no effect on WT mice. Brain sections from 17-20 months old mice of indicated genotypes were imaged at 594nm for auto fluorescent signals (*red*). The auto fluorescent signals were quantified by Image J. Hochest 33324 was used as a marker for nuclei (*blue*). Scale bar=100 μm (**b**, **c**) Quantification of (**a**). *n*=3, student’s t-test, **, p<0.01; ***, p<0.001; ns, no significance

**Fig. 6:**
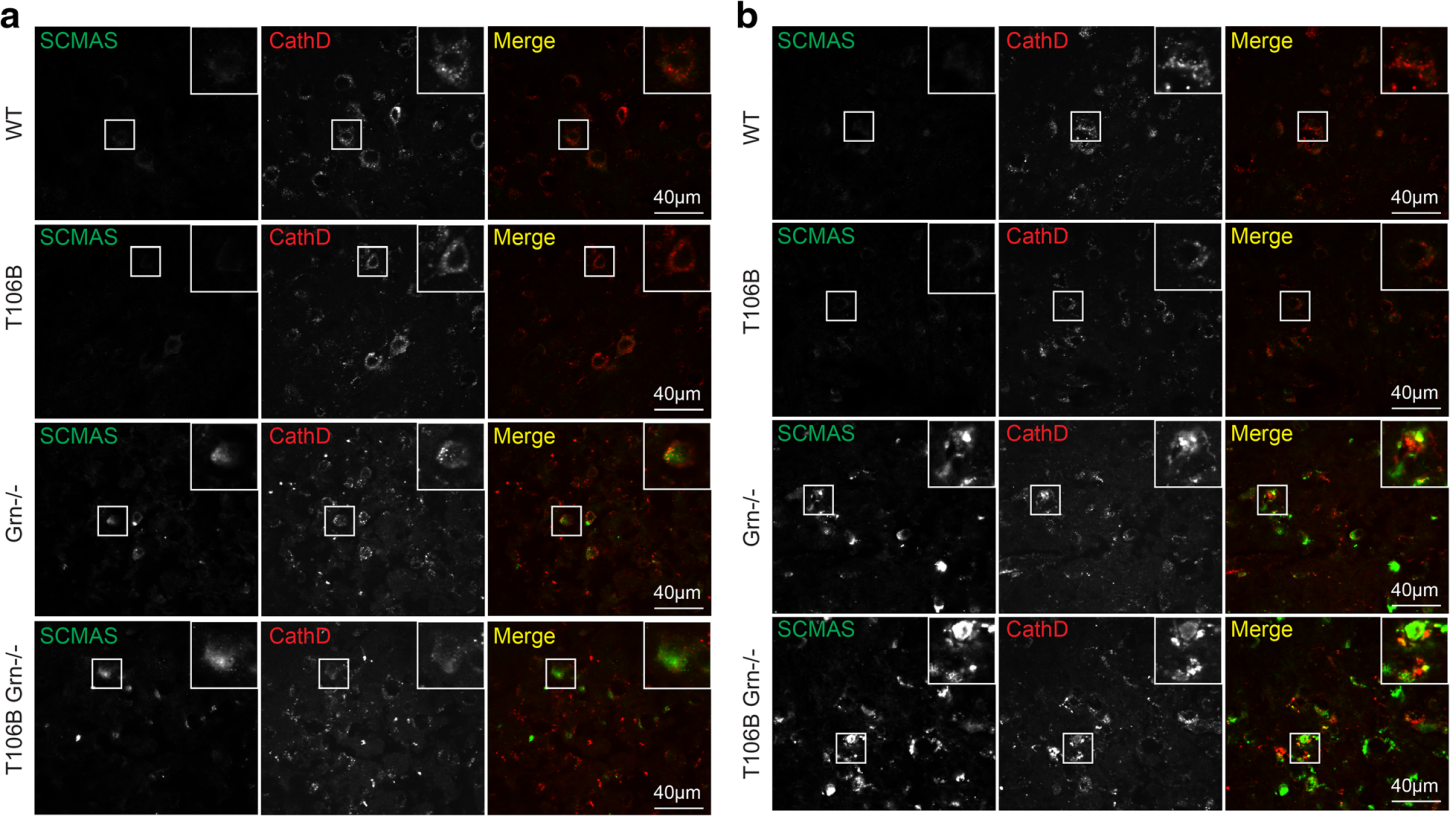
TMEM106B transgene expression increases SCMAS aggregation in PGRN deficient mice. Brain sections (**a**, cortex; **b**, thalamus) from 17-20months old mice of indicated genotypes were stained with anti-SCMAS and anti-cathepsin D antibodies. Scale bar = 40 μm

**Fig. 7:**
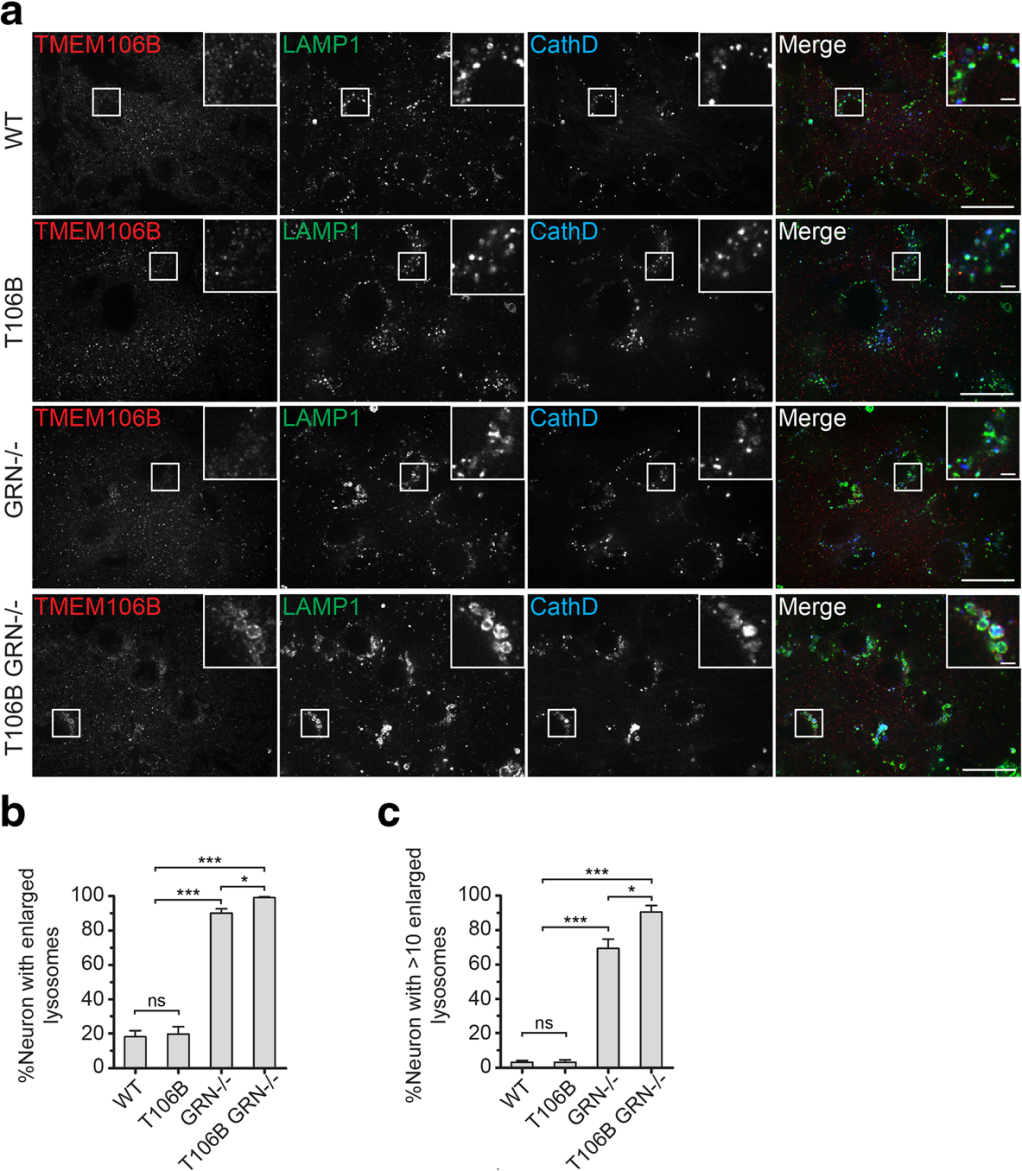
TMEM106B transgene expression exacerbates lysosomal abnormalities in PGRN deficient mice. **a** TMEM106B transgene expression results in more lysosomal enlargement in PGRN−/− mice but has no effect on WT mice. Brain sections from 17-20 month old mice of indicated genotypes were stained with anti-TMEM106B, anti-LAMP1 and anti-cathepsin D antibodies. Neurons from layer III-V cortical regions were shown as examples. Scale bar = 20 μm (inset: 2 μm) (**b**) Percentage of neurons containing enlarged lysosomes (>1 μm) were quantified for experiment in (**a**). **c** Percentage of neurons containing more than 10 enlarged lysosomes (>1 μm) were quantified for experiment in (**a**). *n =* 3, one-way ANOVA, *, *p <* 0.05; ***, *p <* 0.001; ns, no significance

Lipofuscin accumulation is an indicator of lysosomal dysfunction and a hallmark for NCL disease. Increased accumulation of lipofuscin has been reported in NCL patients with PGRN loss [35] and in PGRN−/− mice [1, 38]. Consistent with these previous studies, we observed a significant increase of lipofuscin in both the cortex and thalamus of the aged PGRN−/− mouse brains (Fig. 5a-c). The lipophilic subunit c of the mitochondrial ATP synthase (SCMAS) is a main component of lipofuscin detected in many NCL patients [9], and was found to aggregate in patients with FTLD due to PGRN mutations [18]. In agreement with these reports, we found that increased SCMAS aggregates in aged PGRN deficient mice compared to controls (Fig. 6a, b). To examine lysosomal phenotypes more carefully, we stained brain sections using anti-LAMP1 antibodies. Consistent with previous reports [29], we found that neuronal lysosomes in the adult mouse brain are typically < 1 μm in size and are often situated in a perinuclear position. Normal aging results in occasional enlarged lysosomes (>1 μm) but this phenotype is dramatically exacerbated by PGRN deficiency (Fig. 7a-c). TMEM106B overexpression causes lysosomal enlargement and dysfunction in cell culture [6, 7, 21]. Thus we examined whether increased TMEM106B levels in the PGRN−/− mice due to transgene expression would lead to enhanced lysosomal dysfunction and lipofuscin accumulation. While the expression of TMEM106B transgene does not result in lipofuscin accumulation or any abnormal lysosomal morphology in the wild type mice, it significantly increased the amount of autofluorescence signals as an indicator of lipofuscin deposition (Fig. 5a-c), SCMAS accumulation (Fig. 6a and b) and the percentage of neurons with enlarged lysosomes in the PGRN deficient mice upon aging (Fig. 7a-c). SCMAS signals are colocalized with lysosomal markers LAMP1 and cathepsin D, indicating SCMAS aggregates are in the lysosomes (Additional file 3). Moreover, the levels of TMEM106B proteins seem correlated with the extent of lysosomal enlargement (Fig. 7a). These results clearly demonstrate that elevated TMEM106B levels exacerbate lysosomal pathology caused by PGRN loss and explain how TMEM106B risk alleles resulting in increased TMEM106B levels serve as the risk factor for FTLD with PGRN mutations.

## Discussion

In this study we generated transgenic mice expressing human TMEM106B cDNA under the neuronal specific promoter CamKII. The expression of human transgene was confirmed by qPCR at the RNA level and by mass spectrometry analysis at the protein level. However, despite the expression of the transgene, the total protein level of TMEM106B in the cortex of the young mice is not changed, suggesting a tight regulation of TMEM106B protein levels in neurons. Our data further showed that PGRN is one of the key mechanisms that promotes TMEM106B turnover in the aged brain.

Multiple studies have suggested a critical role of TMEM106B in regulating lysosomal function [6, 7, 20, 21, 36] and elevated TMEM106B levels are associated with increased risk for FTLD-TDP with PGRN mutations [10, 15, 41, 43]. Since proper lysosomal function is critical towards preventing neurodegeneration, it is not surprising that TMEM106B levels are tightly regulated to ensure proper lysosomal activities. Indeed, TMEM106B protein is a substrate for lysosomal degradation [6, 7, 21]. Thus healthy lysosomes are able to maintain proper TMEM106B levels through their own degradative activities.

Loss of PGRN results in NCL in humans [35] and increased accumulation of lipofuscin in mice during aging [1, 38]. We also demonstrated that PGRN is a lysosomal resident protein [19, 46]. However, the precise function of PGRN in the lysosome is still unknown. Our data support that at least one function of PGRN is to promote TMEM106B degradation to maintain the proper level of TMEM106B on lysosomal membranes in the aged brain. How PGRN performs this action, though, remains unknown. One possibility is that PGRN helps maintain the proper activity of lysosomal enzymes during aging. In addition to TMEM106B, other lysosomal substrates accumulate in response to PGRN deficiency. SCMAS and saposin D, components of the lipofuscin, aggregate in PGRN−/− mouse brain and FTLD-PGRN patient samples (Fig. 6) [18]. It remains to be determined whether PGRN plays a direct role in TMEM106B turnover or indirectly by regulating lysosomal functions.

TDP-43 aggregation and hyper-phosphorylation is a hallmark for FTLD with PGRN mutations [4, 11, 17]. However, we failed to detect TDP-43 pathology in our PGRN deficient mice with or without TMEM106B transgene overexpression (data not shown). Thus the FTLD pathology is not fully recapitulated in mouse models.

**Fig. 8:**
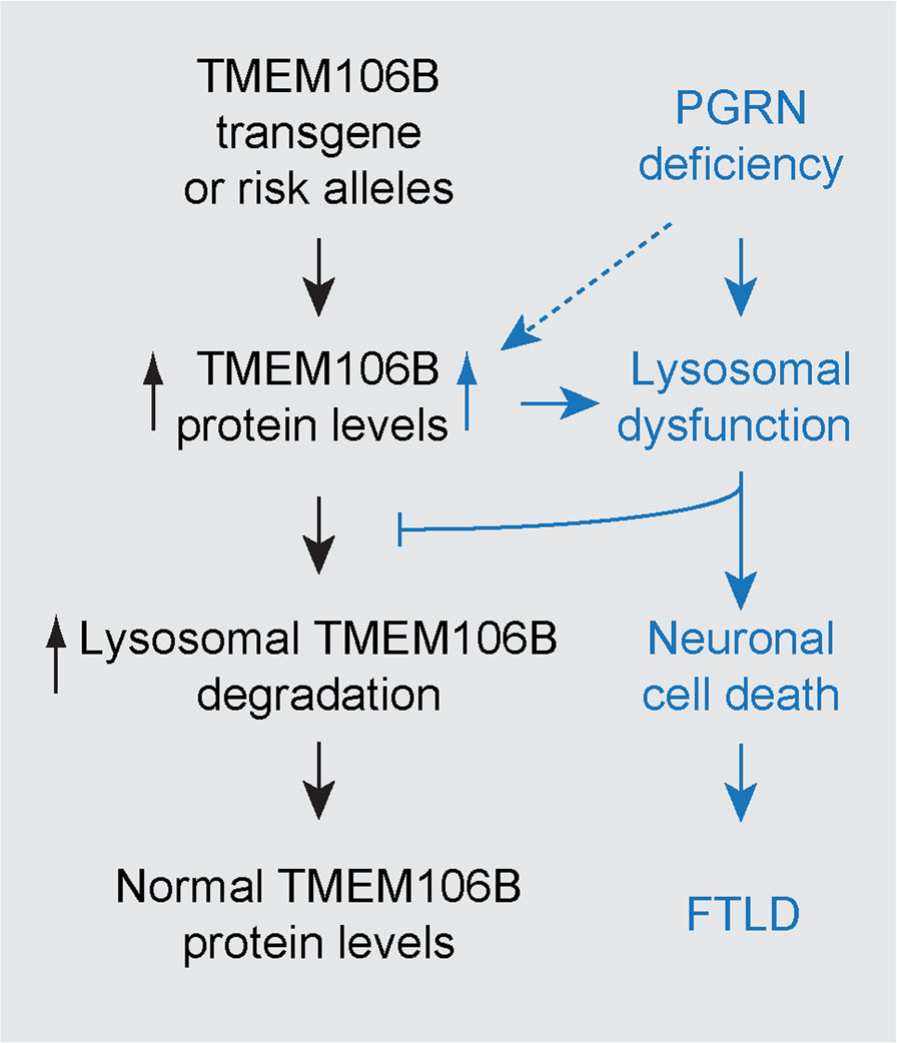
A model for the cross-regulation between TMEM106B and PGRN in the transgenic mouse model and in FTLD/PGRN patients with TMEM106B risk allele. Lysosomal dysfunction caused by PGRN deficiency during aging leads to TMEM106B accumulation, which results in more severe lysosomal pathology and eventually neuronal death in FTLD. PGRN might also play a direct role in regulating TMEM106B levels (*dashed line*)

Nevertheless, we have generated a transgenic model for TMEM106B function in FTLD. Our mouse model closely mimics the interplay between PGRN and TMEM106B during FTLD progression. First, much like in human patients, the increase of TMEM106B protein levels from the transgene expression is much more evident in PGRN deficient mice, suggesting a regulation of TMEM106B levels by PGRN. Second, TMEM106B transgene expression only affects lysosomal morphology and lipofuscin deposition in a PGRN deficient background, closely mimicking human cases in which TMEM106B has been identified as a risk factor for FTLD with PGRN mutations. Third, the effect of PGRN on TMEM106B turnover and proper lysosomal function is much more evident in the aged brain, consistent with notions that aging is the biggest risk factor for neurodegenerative diseases and lysosomal function declines with age. Our data led us to propose a model on the relationship between PGRN, TMEM106B, lysosomes and FTLD (Fig. 8). TMEM106B protein levels are tightly regulated through lysosomal activities. In healthy adults, increased TMEM106B protein production due to the transgene expression or from TMEM106B risk allele is quickly balanced by increased lysosomal degradation of TMEM106B, resulting in normal TMEM106B levels. However, this regulation of TMEM106B levels is disrupted by PGRN mutations, which results in lysosomal dysfunction coupled with aging. Consequently TMEM106B levels are elevated in PGRN deficient background in the aged brain. This increase in TMEM106B levels further exacerbates lysosomal dysfunction, leading to increased lysosomal enlargement and lipofuscin accumulation, and also a further increase in TMEM106B levels. This positive feedback loop eventually leads to loss of lysosomal function and neuronal death with aging.

A portion of FTLD patients also develop amyotrophic lateral sclerosis (ALS) phenotypes (FTLD/ALS). Hexanucleotide repeat expansion in the intron region of the C9orf72 gene, are responsible for the majority cases of FTLD/ALS with TDP-43 aggregates [12, 30, 44]. Recently TMEM106B polymorphisms have been shown to modify the disease phenotypes in FTLD/ALS cases with repeat expansions in the C9orf72 gene [16, 22, 40]. C9orf72 might also be involved in endolysosomal trafficking and autophagy-lysosome pathway [2, 8, 14, 27, 34, 37, 39, 45]. TMEM106B is also implicated in pathological presentation of Alzheimer’s disease [31] and lysosomal dysfunction has been shown to be implicated in Alzheimer’s disease as well. Thus it will be interesting to investigate whether lysosomal impairment in FTLD/ALS/C9orf72 and AD cases could trigger the imbalance in TMEM106B protein homeostasis that leads to TMEM106B induced toxicity.

## Conclusion

Our TMEM106B transgenic mouse model nicely recapitulates the interaction between progranulin and TMEM106B in human patients and support a regulation of TMEM106B by progranulin in the aged brain and a role of TMEM106B in FTLD-PGRN disease progression.

## Additional files

Additional file 1: Western blot to show the heat sensitivity of TMEM106B protein. (PDF 193 kb)
Additional file 2: Sequence alignment of human and mouse TMEM106B proteins. (PDF 667 kb)
Additional file 3: Colocalization of SCMAS with lysosomal marker LAMP1 and cathepsin D in thalamus section of 17 month old TMEM106B Grn−/− mice. (PDF 940 kb)
