# Supplementary material for: Elevated TMEM106B levels exaggerate lipofuscin accumulation and lysosomal dysfunction in aged mice with progranulin deficiency
Source: Acta Neuropathol Commun. 2017 Jan 26;5:9. doi: 10.1186/s40478-017-0412-1 (PMC5270347; doi:10.1186/s40478-017-0412-1)
Supplement: Additional file 1: — Western blot to show the heat sensitivity of TMEM106B protein. (PDF 193 kb) [file 40478_2017_412_MOESM1_ESM.pdf]

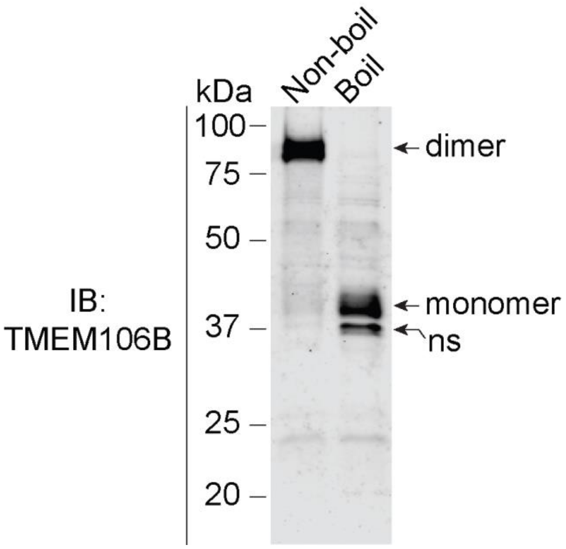

Additional file 1: Western blot of WT mouse brain lysate with anti-TMEM106B antibodies. The lysate in the SDS sample buffer without 2-mercaptoethanol was either kept on ice all the time (non-boiled) or boiled at 95C for 2 minutes (boiled) before loading on SDS-PAGE. Under cold conditions, TMEM106B runs as a dimer that's reduced to monomer upon boiling.
