# Supplementary material for: Elevated TMEM106B levels exaggerate lipofuscin accumulation and lysosomal dysfunction in aged mice with progranulin deficiency
Source: Acta Neuropathol Commun. 2017 Jan 26;5:9. doi: 10.1186/s40478-017-0412-1 (PMC5270347; doi:10.1186/s40478-017-0412-1)
Supplement: Additional file 2: — Sequence alignment of human and mouse TMEM106B proteins. (PDF 667 kb) [file 40478_2017_412_MOESM2_ESM.pdf]

```
mouse  1    MGKSLSHLPLHSNKEDGYDGVTSTDNMRNGLVSSEVHNEDGRNGDVSQFPYVEFTGRDSV  60
            MGKSLSHLPLHS+KED YDGVTS +NMRNGLV+SEVHNEDGRNGDVSQFPYVEFTGRDSV
human  1    MGKSLSHLPLHSSKEDAYDGVTS-ENMRNGLVNSEVHNEDGRNGDVSQFPYVEFTGRDSV  59

mouse  61   TCPTCQGTGRIPRGQENQLVALIPYSDQRLRPRRTKLYVMASVFVCLLLSGLAVFFLFPR  120
            TCPTCQGTGRIPRGQENQLVALIPYSDQRLRPRRTKLYVMASVFVCLLLSGLAVFFLFPR
human  60   TCPTCQGTGRIPRGQENQLVALIPYSDQRLRPRRTKLYVMASVFVCLLLSGLAVFFLFPR  119

mouse  121  SIEVKYIGVKSAYVSYDAEKRTIYLNITNTLNITNNNYYSVEVENITAQVQFSKTVIGKA  180
            SI+VKYIGVKSAYVSYD +KRTIYLNITNTLNITNNNYYSVEVENITAQVQFSKTVIGKA
human  120  SIDVKYIGVKSAYVSYDVQKRTIYLNITNTLNITNNNYYSVEVENITAQVQFSKTVIGKA  179

mouse  181  RLNNITNIGPLDMKQIDYTVPTVIAEEMSYMYDFCTLLSIKVHNIVLMMQVTVTTAYFGH  240
            RLNNIT IGPLDMKQIDYTVPTVIAEEMSYMYDFCTL+SIKVHNIVLMMQVTVTT YFGH
human  180  RLNNITIIGPLDMKQIDYTVPTVIAEEMSYMYDFCTLISIKVHNIVLMMQVTVTTTYFGH  239

mouse  241  SEQISQERYQYVDCGRNTTYQLAQSEYLNVLQPQQ  275
            SEQISQERYQYVDCGRNTTYQL QSEYLNVLQPQ
human  240  SEQISQERYQYVDCGRNTTYQLGQSEYLNVLQPQH  274
```

Additional file 2: Alignment of mouse and human TMEM106B protein sequences.
