# Supplementary material for: Elevated TMEM106B levels exaggerate lipofuscin accumulation and lysosomal dysfunction in aged mice with progranulin deficiency
Source: Acta Neuropathol Commun. 2017 Jan 26;5:9. doi: 10.1186/s40478-017-0412-1 (PMC5270347; doi:10.1186/s40478-017-0412-1)
Supplement: Additional file 3: — Colocalization of SCMAS with lysosomal marker LAMP1 and cathepsin D in thalamus section of 17 month old TMEM106B Grn−/− mice. (PDF 940 kb) [file 40478_2017_412_MOESM3_ESM.pdf]

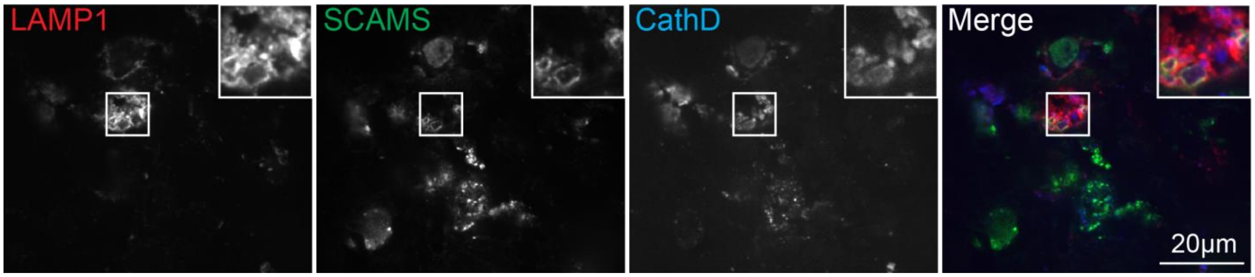

Additional file 3: Colocalization of SCMAS with lysosomal marker LAMP1 and cathepsin D in thalamus section of 17 month old TMEM106B Grn-/- mice. Scale bar=20µm
